# Latest Achievements in the Development of Nanoparticle-Based Drug Delivery Systems of Pt Drugs and Prodrugs in Cancer Therapy

**DOI:** 10.3390/pharmaceutics17101267

**Published:** 2025-09-26

**Authors:** Vlad Iova, Gilda Mihaela Iova, Andreea Taisia Tiron, Ioana Scrobota, Silviu Vlad, Mihail Silviu Tudosie

**Affiliations:** 1 Faculty of Medicine, “Carol Davila” University of Medicine and Pharmacy Bucharest, 020021 Bucharest, Romania; vlad.iova2022@stud.umfcd.ro (V.I.); andreea.tiron@umfcd.ro (A.T.T.); mihail.tudosie@umfcd.ro (M.S.T.); 2 Department of Dental Medicine, Faculty of Medicine and Pharmacy, University of Oradea, 10 1st Decembrie Street, 410073 Oradea, Romania; gilda_iova@uoradea.ro; 3 Department of Medical Semiology, “Sf. Ioan” Emergency Clinical Hospital, “Carol Davila” University of Medicine and Pharmacy, 042122 Bucharest, Romania; 4 Department of Surgical Specialties, Faculty of Medicine and Pharmacy, University of Oradea, 10 1st Decembrie Street, 410073 Oradea, Romania; silviu.vlad@didactic.uoradea.ro; 5 ICU II Toxicology, Clinical Emergency Hospital, 014461 Bucharest, Romania

**Keywords:** nanomedicine, cancer, Pt drugs, drug delivery systems, nanoplatforms

## Abstract

Even though Pt(II)-based drugs represent the standard in cancer therapy, their use is seriously limited by severe side-effects (renal toxicity, allergic reactions, gastrointestinal disorders, hemorrhage and hearing loss), drug resistance and a grim prognosis. This review presents the results of multiple studies showing different nanoparticle-based platforms as delivery agents in order to overcome these drawbacks. The approach of using nanoparticle-based drug delivery systems of Pt drugs and prodrugs is promising due to key advantages like specific targeting and thereby reduced toxicity to healthy cells; increased stability in the bloodstream; multiple mechanisms of action such as stimulating anti-tumor immunity, responding to environmental stimuli (light, pH, etc.), or penetrating deeper into tissues; enhanced efficacy by their combination with other therapies (chemotherapy, gene therapy) to amplify the anti-tumor effect. However, certain challenges need to be overcome before these solutions can be widely applied in clinics. These include issues related to biocompatibility, large-scale production, and regulatory approvals. In conclusion, using nanoparticles to deliver Pt-based drugs represents an advanced and highly promising strategy to make chemotherapy more effective and less toxic. Nonetheless, further studies are required for the better understanding of intracellular mechanisms of action, toxicity and the pharmacokinetics of nanoparticles, and physical–chemical standardization.

## 1. Introduction

Transition metal complex-based medicine represents a promising approach for different therapies due to their wide range of actions (anti-inflammatory, antibacterial, antifungal, anticancer), as they can interact with various biological targets, such as deoxyribonucleic acid (DNA) or enzymes, and can induce the formation of reactive oxygen species (ROS) [1]. As a result, researchers seek to discover new metal-based anticancer drugs like Pd(II)-based ones, with lower side-effects. However, Pt(II)-based drugs continue to represent the standard in cancer therapy [1]. The synthesis of cisplatin, cis-[Pt(II)(NH_3_)_2_Cl_2_] ([PtCl_2_(NH_3_)_2_] or CDDP), a Pt(II) complex, was an important discovery representing proof of Pt(II)-containing compounds’ potential as anticancer drugs and encouraged the study of other transition metal-containing complexes [2,3]. However, their use in different schemes is seriously limited by severe side-effects, drug resistance and a grim prognosis [4]. Consequently, the research subject moved to Pt(IV)-based prodrugs, which can exert their anticancer effects after reduction to their Pt(II) counterparts, having fewer side-effects. Therefore, several Pt(IV) compounds, like ormaplatin or tetraplatin, iproplatin and oxoplatin, are the subject of current research [5,6,7]. To overcome conventional Pt-based drug impediments, the usage of supramolecular chemistry was proposed to produce novel delivery platforms integrating the biological properties of Pt compounds and new supramolecular structures, such as nanoparticle (NP)-based delivery systems [1,8]. Besides NP-based delivery and prodrug methods, ligand modification strategies were employed to increase the selectivity of anticancer agents and reduce their side-effects [1]. NP-based drug delivery platforms seem to deliver Pt(IV) prodrugs more selectively and safely to cancer cells, but clinical outcomes were nevertheless unpredictable because of insufficient in vivo pharmacokinetics studies [9].

In this work, we aimed to present some of the most recent research in the field of nanoparticle-based drug delivery systems of Pt anticancer drugs, focusing on their structure, pharmacokinetics, mechanism of action and alternatives of reducing the severe side-effects of conventional cancer therapy.

## 2. Conventional Pt(II)-Based Cancer Therapy and Its Limitations

**Figure 1:**
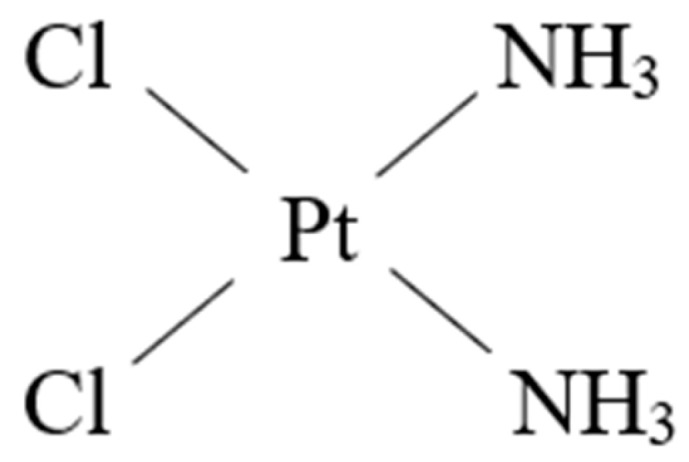
The structure of cisplatin.

Ever since cisplatin (Figure 1) received approval from the U.S. Food and Drug Administration (FDA) for cancer therapy in the 1970s, Pt-based anticancer drugs have become the standard of chemotherapy treatments. However, their wider use is highly limited due to severe side-effects and drug resistance [10].

Cisplatin has been used for the therapy of an array of human tumors like bladder, head and neck, lung, ovarian, and testicular cancers, and against various types of cancers, including carcinomas, germ cell tumors, lymphomas, and sarcomas [11]. It can induce cancer cell death by crosslinking with the purine bases on the DNA, interfering with DNA repair processes, eventually leading to DNA damage [11].

Even though cisplatin has been used for the treatment of numerous human cancers and is effective against various types of cancers, its use is limited by drug resistance and numerous undesirable side-effects such as severe kidney problems, allergic reactions, decreased immunity to infections, gastrointestinal disorders, hemorrhage, and hearing loss especially in younger patients.

The mechanisms of their production and the associated complications and symptoms are depicted in Table 1.

**Figure 2:**
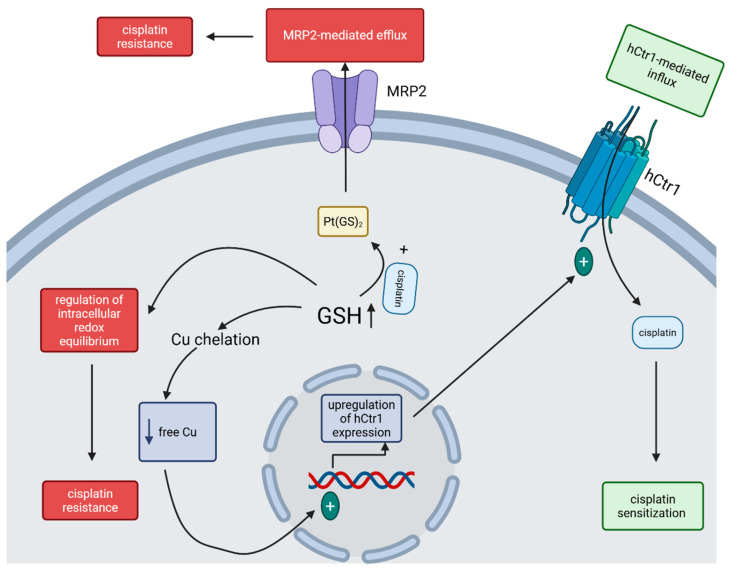
Mechanisms of GSH-regulated cisplatin sensitivity of tumor cells. The increased GSH level inside the cancer cell can, on one hand, enhance the MRP2-mediated efflux of cisplatin and regulate the intracellular redox equilibrium, leading to cisplatin resistance, and, on the other hand, lower the level of free Cu within the cell, upregulate the expression of hCtr1, increase the hCtr1-mediated cisplatin influx and lead to sensitization to cisplatin. (Created in BioRender. Iova, V. (2025) https://BioRender.com/wys8gdv, accessed on 23 July 2025).

Developing drug resistance is common in cisplatin therapy, and it can occur due to high thiol-containing species (especially glutathione (GSH)) levels within the cancer cell and adenosine triphosphate (ATP)-dependent GSH S-conjugate pumps [20]. Several processes are considered in explaining the influence of GSH on cisplatin transport: GSH may function as a cofactor in the ATP-dependent MRP2-mediated efflux of Pt(GS)_2_—a conjugate formed between cisplatin and elevated intracellular GSH—thereby contributing to cisplatin resistance in cancer cells; GSH acts as a cytoprotective agent—highly elevated in cancer cells due to the enhanced activity of γ-glutamylcysteine synthetase (the rate-limiting enzyme in GSH synthesis) and therefore being able to regulate the intracellular redox equilibrium, which contributes to cisplatin resistance; high levels of GSH, by chelating Cu, decreasing Cu levels within cells, and can therefore induce the overexpression of the high-affinity Cu transporter (hCtr1) which also functions as a cisplatin transporter—by mediating cisplatin uptake, its sensitivity to this drug is increased (Figure 2) [21,22].

The importance of each of these mechanisms might depend on the specific type of cancer cell and its physiological state. While ATP-dependent MRP2-mediated efflux of cisplatin requires GSH, Pt(GS)2 formation as an important step for cisplatin elimination remains somewhat controversial as different studies show different results [21]. Additionally, cisplatin can induce the upregulation of γ-GCSh and MRP, thus enhancing the cisplatin efflux [21]. Nevertheless, transfection of recombinant DNA coding only for the γ-GCSh subunit leads to an increase in GSH levels in the transfected cells, with a surprising hypersensitivity instead of resistance to cisplatin toxicity, which was proved to be linked with the enhanced uptake of cisplatin in these transfected cells through the upregulation of hCtr1 [21]. Future investigations are needed to clearly demonstrate the role of GSH in regulating the cisplatin sensitivity of cancer cells.

## 3. Pt(IV) Anticancer Prodrugs

Pt(IV) compounds were synthesized and preclinically (mostly only in vitro) tested to counteract the severe side-effects and drug resistance associated with classical Pt(II) compounds [23]. The octahedral Pt(IV) compounds, that are kinetically inactive, were developed using different synthesis techniques such as conjugation with lipid chains to increase lipophilicity; combination with small peptide segments or NPs to increase drug delivery efficacy; incorporation of bioactive ligands to the axial positions of Pt(IV) compounds for dual-function effects, such as histone deacetylase inhibitors, p53 agonists, alkylating agents, and nonsteroidal anti-inflammatory molecules—molecules that are released after the reduction reaction of the prodrugs to their corresponding Pt(II) active counterparts [24,25,26,27]. This reduction process occurs within the cancer cell due to the redox disequilibrium at the high metabolic rate, increased mitochondrial dysfunction, highly activated cell signaling pathways and fast peroxisomal activities [26,27].

## 4. Nanoparticle-Based Drug Delivery Systems of Pt Drugs in Cancer Therapy

There are many research directions regarding the next generation Pt chemotherapy and the NP-based delivery platforms of Pt complexes (such as various Pt-polymer complexes, micelles, dendrimers and liposomes) providing promising preclinical and clinical results [28]. Several properties and mechanisms of action of NPs encapsulating Pt drugs were studied (Table 2).

There are many biological models used to assess the performance of NPs delivering Pt-based drugs or prodrugs, such as cancer cell lines (for example, MCF-7, HeLa and A549), mouse xenograft models, syngeneic mouse models or patient-derived xenografts [29,30,31]. For the approval of Pt-based nanoformulations in clinical practice, further research and clinical validation are required to confirm the promising results of preclinical studies. Different platforms, such as lipid-based NPs, polymeric NPs, inorganic NPs, and extracellular vesicles were proved to provide increased drug targeting, bioavailability, reduced systemic toxicity in preclinical and clinical studies, the capacity to reverse drug resistance and the potential to be used in combination therapy. However, problems linked to biocompatibility, scalability, and regulatory approval need to be solved for widespread clinical adoption [32].

### 4.1. Strategies Used for a More Efficient Cancer Treatment

#### 4.1.1. Mitochondria Targeting

It was thought and further demonstrated that anticancer drugs targeting mitochondria could potentially overcome or reverse cisplatin resistance [33]. Mitochondria have a central place in the study of tumoral resistance to cisplatin for two reasons. Firstly, the reprogramming and variability of tumor metabolism contributes to cisplatin resistance in cancer cells by enabling a switch between mitochondrial oxidative phosphorylation and glycolysis. Secondly, mitochondrial DNA is more prone to being damaged by Pt-based drugs, and this results in affected mitochondrial oxidative phosphorylation [33].

The latest achievements in mitochondria-targeting are pointing to a self-assembled drug delivery nanoplatform formed by lonidamine (LND)-S-S-Pt-triphenylphosphine (TPP)/β-cyclodextrin-grafted hyaluronic acid (HA-CD) synthesized to destroy cisplatin-resistant lung cancer cells, with every component comprising the nanomolecule bearing an important role: HA-CD can find the CD44 receptor on the tumor cell membrane; TPP may target the mitochondria, where the disulfide bond linking LND and the Pt(IV) prodrug is broken by the high levels of GSH, leading to the release of LND that can inhibit glycolysis and damage these organelles; the Pt(IV) molecule is reduced to cisplatin by GSH and further causes mitochondrial damage by targeting the mitochondrial DNA (mtDNA). By decreasing the intracellular level of GSH, this nanoplatform overcame GSH-mediated chemotherapy resistance [33].

Another recent method for targeting mitochondria is represented by the linkage of oxoplatin with lithocholic acid that self-assemble in water to spherical-shaped NPs. Their mechanisms of action might require the reduction in the Pt(IV) core to Pt(II) and simultaneous release of lithocholic acid, both being important for the anticancer effect. It was showed that the complex with the greatest potency was the one bearing a heptanoate rest linked to the Pt(IV) core at the axial trans position to the lithocholic acid rest [34]. It can affect the cancer cell by various mechanisms: halting the cell cycle in the S and G2 phases, affecting DNA, disrupting mitochondrial membrane potential, increasing ROS production, affecting mitochondrial bioenergetics in PC3 cells associated with human prostate cancer, upregulating pro-apoptotic proteins and reducing anti-apoptotic ones from the B-cell lymphoma 2 (BCl-2) family [34,35,36] (Table 3).

**Figure 3:**
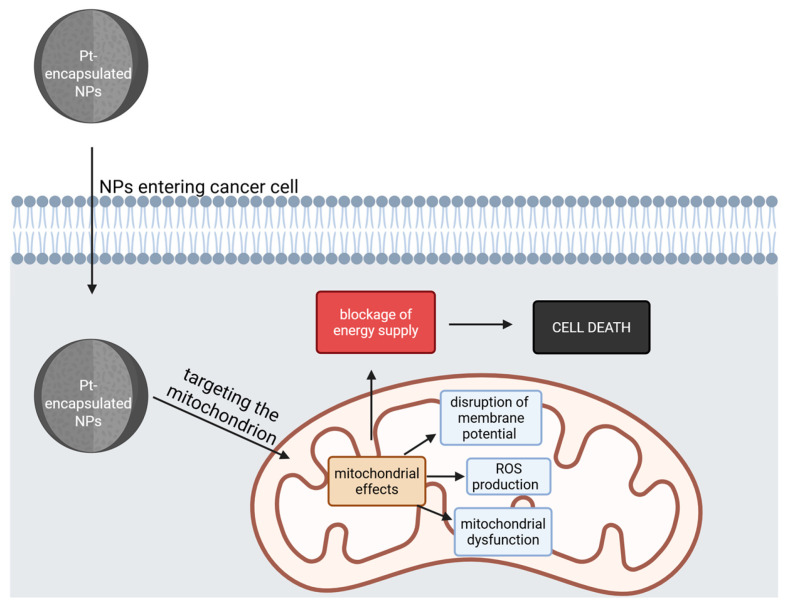
The general effects on mitochondria of Pt-encapsulated NPs. After entering the cancer cell, the NPs target and damage the mitochondria, leading to a blockage of energy supply, causing cell death (Created with BioRender https://biorender.com/0hj2h8q, accessed on 1 august 2025).

The general mitochondrial effects of these compounds are depicted in Figure 3.

#### 4.1.2. Increased Blood Stability

**Figure 4:**
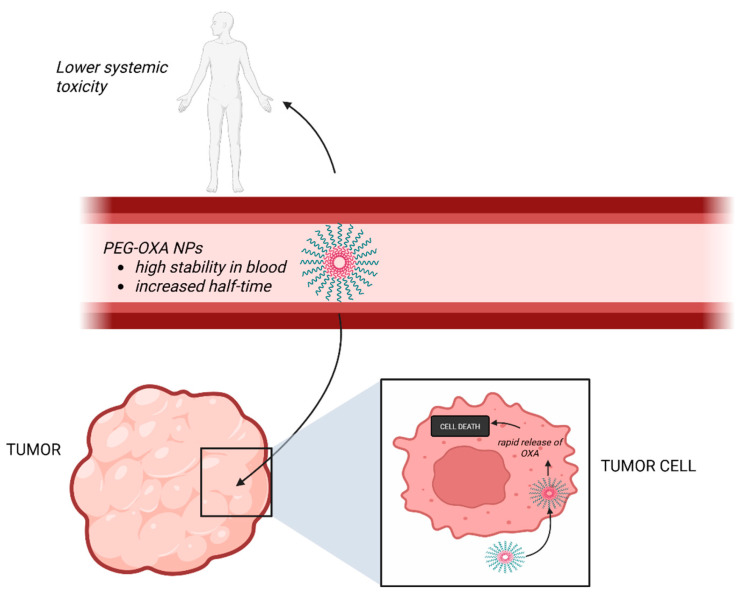
The effects of PEG-OXA NPs. These NPs have a high stability in the blood, with an enhanced circulation time, lower systemic toxicity and increased anti-tumor efficacy. (Created with BioRender https://BioRender.com/s4pnyfr, accessed on 1 august 2025).

The study of pharmacokinetics is pivotal for the understanding of drug therapeutic potential. Optimizing absorption, delivery and elimination of Pt-based anticancer drugs is essential to increase antitumor efficacy, decrease side-effects and enhance inhibition of the growing of tumors, and a type of NPs, designated PEG-OXA NPs, appear to exhibit these characteristics [9]. PEG-OXA NPs are formed from a new Pt(IV) complex derived from oxaliplatin (OXA), polyethylene glycol (PEG)-OXA, respectively, that has two hydrophobic lipid rests and the hydrophilic PEG in axial positions and can further self-assemble in micellar NPs in an aqueous environment [9]. These NPs have raised the interest of researchers for they seem to facilitate a rapid release of bioactive OXA in tumor cells and manifest a high stability in blood in vitro and an increased half-life in vivo, thus providing an emerging solution for anticancer drug-targeted delivery [9] (Figure 4).

Besides the novel proposal of PEG-OXA NPs, other recent approaches including nanocrystals, self-assembled PtNPs with protein coronas, nano-hydrogels and mesoporous silica nanoparticles (MSN) are depicted in Table 4.

#### 4.1.3. Increased Anti-Tumoral Immunity

One study described a novel NP-based delivery platform for the targeted delivery of Pt(IV) containing a trisulfide bond, which exhibited increased in vivo anticancer properties and reduced liver damage (compared to cisplatin), namely NP(3S)s [41]. Due to high GSH levels within tumor cells, these compounds might release active Pt(II) and hydrogen sulfide (H_2_S) that synergistically act together by impacting DNA (the classical mechanism of Pt(II)); activating the stimulator of interferon gene (STING) pathway; activating T cells and subsequently increasing antitumor immunity; and activating the OS mechanisms [41].

Another efficient combination of Pt-based chemotherapy and immunotherapy was shown for osteosarcoma, a type of cancer where most immune checkpoint blockades were not proved to have good results. For instance, a type of NP designated as NP-Pt-IDOi presented a great antitumor effect [42]. NP-Pt-IDOi was synthesized from a ROS-responsive amphiphilic polymer containing thiol-ketal bonds within its structure by co-encapsulating both a Pt(IV) prodrug and an indoleamine-(2/3)-dioxygenase inhibitor (IDOi) [42]. After entering an environment with high levels of ROS, such as the one in cancer cells, the NP released both encapsulated molecules. The Pt(IV) prodrug was reduced to its Pt(II) counterpart, thus damaging DNA and inducing the STING pathway, with a subsequent increase in antitumoral immunity. The concomitantly released IDOi inhibited the metabolism of tryptophan and, consequently, further activated the cytotoxic CD8+ T cells and enhanced the anticancer effects of the nanostructure [42]. Moreover, another way to increase therapy outcome for osteosarcoma by increasing the targeting capacity of the drug and decreasing or reversing the tumor-associated immunosuppression was proved to be achieved by the self-assembly of an OXA-based Pt(IV) prodrug amphiphile, namely Lipo-OXA-ALN, in which alendronate (ALN) acts as a targeting agent for osteosarcoma cells [43]. The effects of the alleged NPs were an enhanced intracellular uptake of OXA, inhibition of cancer cell activity, increased targeting capacity (to spare the healthy bone), increased antitumoral immunity, increased M1/M2 macrophage ratio and modification in the tumoral microenvironment [43].

**Figure 5:**
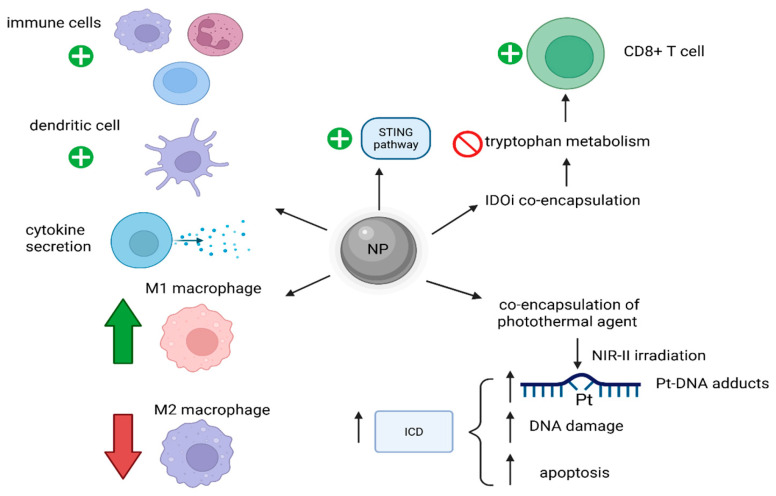
Mechanisms of NP-induced increased anti-tumoral immunity. These NPs activate immune cells, increase cytokine secretion, increase the M1/M2 macrophage ratio and can enhance ICD. (Created with BioRender https://BioRender.com/4ffd2r8, accessed on 1 august 2025).

A further effect of Pt-based drugs, like OXA, is that they can induce immunogenic cell death (ICD) in cancer cells by activating the immune system, and, as a result, strategies to enhance these ICD effects were studied [44]. As a result, it was proved that an NP-encapsulating OXA and a near-infrared-II (NIR-II) photothermal agent IR1061 could achieve this enhancement of ICD in tumors after irradiation with NIR-II, leading to a mild increase in temperature, but with important effects. Increased Pt-DNA binding, higher DNA damage and apoptosis, with subsequent stronger ICD were reported [44]. Such a strategy remarkably increased the therapy results for triple-negative breast cancer 4T1 in comparison with either OXA or NIR-II-based photothermal therapies alone (Figure 5) [44].

Current research focuses on Pt-conjugated iron NPs, hyperthermia sensitive Pt NPs and immunogenic bifunctional nanoparticles, as well (Table 5).

#### 4.1.4. Multistimuli-Responsive Drug Delivery Systems

To overcome the drawbacks of conventional Pt(II) chemotherapy, stimuli-responsive nanoplatform-based delivery systems were considered due to their great potential for exact spatiotemporal drug deliverance [48]. Multistimuli-responsive drug delivery systems are employed to specifically target the site of a drug’s action and release the therapeutic agent by responding to multiple internal and external signals, such as electrical and magnetic fields, acidity, temperature, light irradiation, and redox stimuli [49].

pH-mediated drug delivery methods are on the rise as promising anticancer therapeutic approaches [50]. Liposomes formed from lipid-encapsulated CaCO_3_ (representing the acidic pH-responsive element of the liposomes) carrying a Pt(IV) prodrug and biotin were proved efficient against thyroid cancer cells, posing high stability and rapid pH-mediated degradation, which, consequently, may direct the pH-responsive delivery of both anticancer molecules [50]. The intracellular effects within the cancer cells of these NPs are increased OS, mitochondrial damage, and DNA damage, ultimately leading to cancer cell death [50].

Another stimuli-responsive approach relies on activation upon irradiation and reduction [48]. Some upconversion NP-based nanoplatforms can incorporate, through hydrophobic interactions, an octahedral Pt(IV) prodrug with an octadecyl aliphatic chain and phenylbutyric acid (i.e., a histone deacetylase inhibitor, with additional anticancer effects) as axial ligands. By further modifications through the binding of 1,2-distearoyl-sn-glycero-3-phosphoethanolamine polyethylene glycol (PEG) 2000 and the peptide arginine-glycine-aspartic, an additional increase in tumor specificity and reduction in systemic toxic effects occur. After upconversion luminescence irradiation and GSH reduction, the NPs release the Pt(II) active counterparts and phenylbutyric acid in the cancer cell cytoplasm, leading to tumor cell death [48].

Synthesis of NPs with both photo- and pH-sensitivity as an encouraging anticancer therapeutic approach was attempted [51]. One such NP is formed from a Pt(IV) prodrug, which can be activated to its Pt(II) active counterpart by ultraviolet A (UVA) irradiation, and demethylcantharidin. Being an inhibitor of protein phosphatase 2A, demethylcantharidin released from the NP in an acidic medium increases the formation of hyper-phosphorylated protein kinase B (pAkt) to halt the DNA repair mechanisms [51,52]. Additionally, a metallo-nano prodrug was developed by attaching a photosensitizer-conjugated Pt(IV) complex to a polymeric core and chelating it with iron. In an acidic microenvironment, iron released from the NP allows the reduction in Pt(IV) to Pt(II) after light irradiation, leading to chemotherapy and photodynamic therapy [40]. Besides these effects, the NP is supposed to cause ferroptosis and tumor-associated macrophage polarization with additional anticancer consequences [53].

A recent study described a nanozyme-based photocatalytic method for Pt(IV) prodrug activation [54]. The authors reported that an AuNP covered by thiol ligands with 1,4,7-triazacyclononane headgroups encapsulating riboflavin-5′-phosphate (FMN) promoted the photocatalytic reduction in a Pt(IV) prodrug to cisplatin in the presence of a reducing agent [54]. Another current approach employs micellar NPs delivering Pt(IV) prodrugs with photosensitivity which, upon UVA irradiation, show enhanced cytotoxicity towards cancer cells, with reduced side-effects and an increased circulation time [55]. Moreover, two recent studies have brought attention to the development of compounds that can be selectively activated upon irradiation and provide spatial and temporal control over the treatment [56]. In this regard Pt(IV) prodrug NPs that could be activated by deeply penetrating ultrasound radiation are considered as a promising alternative to the common activation of Pt(IV) prodrugs through ultraviolet or blue light irradiation that can reach only the surface, being ineffective against deep or very thick tumors [56] A recent study showed that self-assembled P-Rf/cisPt(IV) NPs transporting a Pt(IV) prodrug and riboflavin, displayed enhanced anti-tumoral properties through an increased activation of the prodrug upon low-intensity ultrasound radiation through superoxide anions, whose formation was increased by riboflavin [57].

Stimuli-responsive techniques might also be employed to overcome resistance to conventional chemotherapy [58]. NPs containing both a Pt(IV) prodrug and ursolic acid known for a higher circulation time, tumor accumulation and antitumor activity but without the side-effects associated with conventional therapy, were proved to reverse cisplatin resistance in ovarian cancer in an intracellular reductive and acidic microenvironment through both molecules released [58]. Self-assembled dual-drug polymer micellar NPs and Pt-coordinated dual-responsive nanogels have gained interest as potential anticancer drugs as well (Table 6).

**Figure 6:**
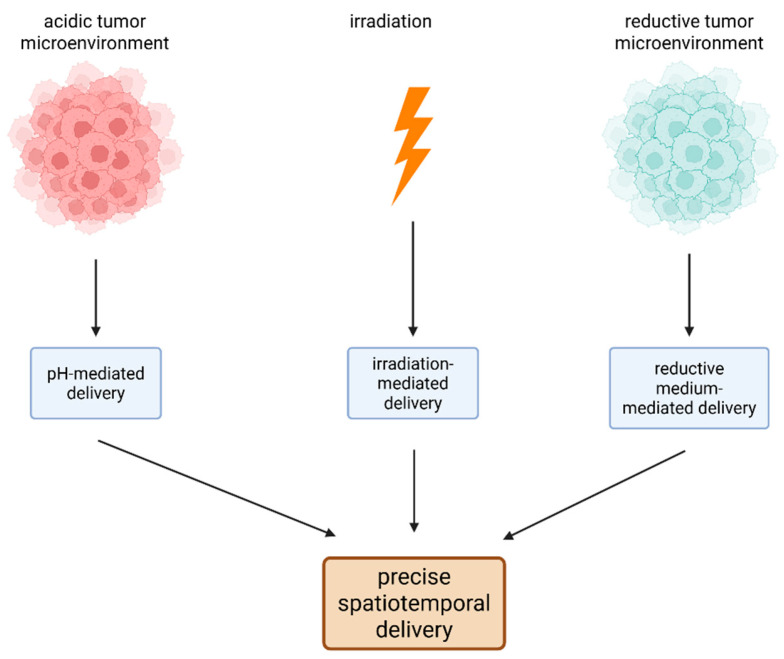
The principles of multistimuli-responsive drug delivery systems of Pt drugs. Because of the special characteristics of the tumor microenvironment (low pH and an increased level of reductive species) and of the NPs (photosensitivity), precise spatiotemporal delivery of anticancer molecules can occur. (Created with BioRender https://BioRender.com/62axkhb, accessed on 1 august 2025).

The principles of multistimuli-responsive drug delivery systems are presented in Figure 6.

#### 4.1.5. Combination Chemotherapy

Nanotechnology-facilitated combinational delivery of anticancer molecules represents a new promising strategy in tumor therapy [60]. Several compounds co-delivered with Pt-based drugs and prodrugs through nanoplatforms were proposed.

Adding Fe compounds to induce the intracellular cascade reaction and generate sufficient HO^•^ for ferroptosis therapy is of great interest [4,61,62,63,64]. H_2_O_2_ depletion-mediated tumor anti-angiogenesis, apoptosis and ferroptosis were found to be achieved by a Pt(IV) prodrug-delivery nanoplatform coated by ferric oxide, which protects the NP in circulation, enhancing delivery to tumor cells, with a selenium core [61]. Several cytotoxic mechanisms on cancer cells were described: ferroptosis caused by HO^•^ accumulation from H_2_O_2_ induced by Fe(II) in acidic cancerous medium; inactivation of glutathione peroxidase 4 (GPX4); OS augmentation and enhancement of ferroptosis as additional effects of Pt(IV)-reduction to Pt(II) complexes; inhibition of angiogenesis resulting from lower levels of vascular endothelial growth factor-A (VEGF-A); mitochondrial damage and affected angiogenesis caused by the exposed selenium core [61]. Polypeptide vehicles carrying cisplatin and Fe_3_O_4_ NPs were synthesized. They were employed as theranostic agents for combination therapy guided by T_2_-weighted magnetic resonance imaging (MRI). The ferroptosis, caused by the HO^•^ mechanism mentioned above, was implied with the difference that Fe_3_O_4_ NPs can also be used for the T_2_-weighted MRI of the tumor [62]. Another study proposed the synthesis of a GSH-responsive NP containing a disulfide bond-based amphiphilic polyphenol, a dopamine-modified cisplatin prodrug and Fe(III) integrated through coordination reactions between Fe(III) and phenols. Increased OS led to ICD, promoting the maturation of dendritic cells and finally enhancing the antitumor immune response [4].

One promising way for targeted drug delivery might be the conjugation of monoclonal antibodies to different nanosystems. As such, cetuximab was used to decorate a Pt-based drug delivery nanoplatform activated upon NIR irradiation. Cetuximab attachment resulted in a nanosystem capable of specific delivery of drugs to epidermal growth factor receptor (EGFR)-hyperexpressing cancer cells in epidermoid carcinoma [65].

In addition, nanoformulation-based drug delivery systems of Pt(IV) prodrugs, like OXA, functionalized with axial ligands represented by other chemotherapeutic agents, like gemcitabine or capecitabine, were proposed. The additional drugs were released upon reduction within cancerous cells with synergistic antitumoral effects and were proved effective in a representative colorectal cancer cell model, with the complexes using gemcitabine being the most active [66].

Multiple studies proposed the association of Pt-based chemotherapy with phototherapy and radiotherapy [20,67,68,69]. The first obvious additional effect besides the therapeutic one is the possibility of simultaneously monitoring the delivery of the Pt-prodrugs by bioimaging [67,68,70]. When synthesis of NPs containing both a Pt(IV) prodrug and a mitochondria-targeting NIR photosensitizer, namely IR780, was developed, it was found that the NIR laser irradiation led to the inhibition of hyperactive energy-generating processes of mitochondria through both photothermal and photodynamic mechanisms, leading to a hindrance of processes requiring energy in the form of ATP, like drug efflux out of the cancer cells or repair of damaged nuclear acids [67,71]. As a result, decrease in pivotal proteins of the nucleotide excision repair pathway activity was induced, thereby enhancing the effects of the Pt-based drugs on the cancer cell’s nucleic acids [67]. Moreover, these NPs allow drug delivery and treatment monitoring through NIR fluorescence and photoacoustic imaging [67]. In another study, Pt(IV)-based polymeric prodrug PVPt with amphiphilic properties was used to encapsulate a theranostic agent, the modified cyanine dye 1-(2-hydroxyethyl)-2-((E)-2-((E)-3-((E)-2-(1-(2-hydroxyethyl)-3,3-dimethylindolin-2-ylidene)ethylidene)-2-chlorocyclohex-1-en-1-ly)vinyl)-3,3-dimethyl-3H-indol-1-ium bromide (HOCyOH or Cy), respectively, through hydrophobic interactions, resulting in NPs formed by self-assembly [68]. These NPs could undergo disassembly and activation under acidic, reductive conditions and NIR laser irradiation, being accompanied by photothermal conversion and increased OS [68]. Regarding the association with radiotherapy, researchers are concentrating on new, safe, and redox-responsive NPs with a higher disulfide density and a better ability to load Pt(IV) prodrugs. These NPs would be useful in reversing cisplatin resistance and improving anticancer outcome by directing more cytotoxic compounds towards tumor cells, scavenging GSH, and causing mitochondrial damage, which would increase Pt-DNA cross-linking and make these cancer cells more sensitive to X-ray radiation. As a result, they are considered promising candidates for anticancer chemoradiotherapy, particularly for cervical cancers [20].

Breaking the redox balance is a promising way to maximize the efficacy of conventional Pt-based cancer therapy because the anticancer activity and drug resistance of chemotherapy are related to the redox state of tumor cells [72]. Therefore, NPs formed from polymers containing a diselenium bond in the main chain surrounding a Pt(IV) prodrug were synthesized. They depleted GSH while increasing the level of OS at the same time, thereby disrupting intracellular redox balance and increasing the antitumoral effect of conventional cisplatin therapy [72].

Additionally, the association of Pt(IV) prodrugs and a ubiquitin-specific protease 1 inhibitor (USP1i) within NPs was found to have an enhanced antitumoral effect in comparison with conventional therapy. USP1i seems to affect the DNA damage repair processes by targeting USP1 to increase the cytotoxic effects of cisplatin against cancer cells [31].

It is widely known that doxorubicin, along with other agents, causes hypoxia-induced multi-drug resistance, resulting in poor therapy results [73]. This process may be reversed and stopped by NPs formed as a co-self-assembly of a PEG-Pt(IV) prodrug and doxorubicin which, under light irradiation, can lead to the generation of oxygen to reverse tumor hypoxia and release active Pt(II) and doxorubicin. Increased OS and enhanced cytotoxicity effects compared to both therapies alone were reported [73].

Another approach combines in NPs an OXA-derived Pt(IV) prodrug and a peptide that targets mitochondria aiming to impact the molecular processes that need energy in the form of ATP [71]. These NPs are supposed to become activated by the acidic tumor microenvironment and increased concentration of GSH within cancer cells, leading to the accumulation of high intracellular levels of Pt-drugs and increased anti-tumoral effects, respectively [71].

Molecules to assess the therapeutic efficacy of the treatment were added besides anticancer drugs and drug tracers [74]. Such NPs were developed from a Pt(IV) compound, a NIR-II fluorophore tracer and a peptide that may be split by caspase-3 and serve as an apoptosis indicator. The combination is considered a promising strategy to determine both the drug pharmacokinetics and its therapeutic success [74].

One strategy potentially effective against cisplatin-resistant tumors might be the synthesis of NPs delivering not only the Pt drug, but also curcumin [75]. This co-delivery platform enhances stability, as well as solubility of curcumin and, additionally, release of the Pt drug and the antioxidant occur in reductive environments, such as those from tumors, with consequent synergistic effects [75] (Table 7).

**Figure 7:**
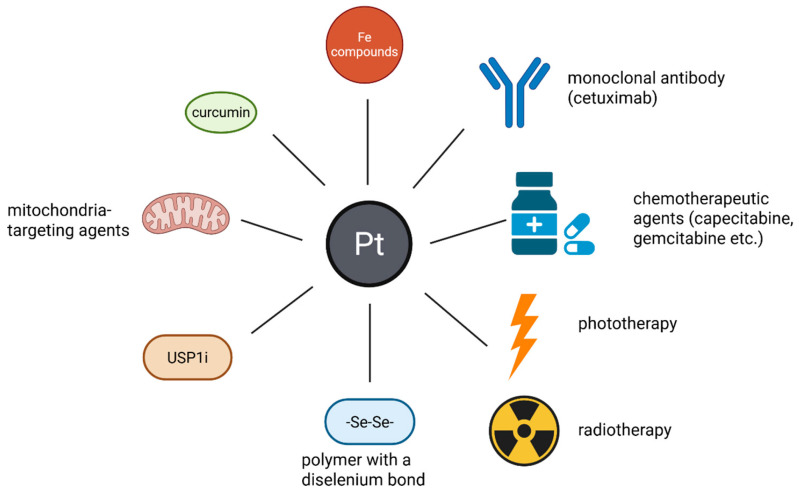
The association of Pt drugs/prodrugs with other compounds or techniques in NP-based combination therapy. These Pt compounds can be associated with other elements (Fe compounds or selenium) monoclonal antibodies (cetuximab), other anti-tumor therapies (chemotherapeutic agents—capecitabine, gemcitabine-phototherapy and radiotherapy) mitochondria targeting agents, USP1i and antioxidants (curcumin). These combinations result in an improved anticancer effect, lower systemic toxicity and the possibility to reverse the resistance of tumors to drugs. (Created with BioRender https://BioRender.com/xyg5ul1, accessed on 1 august 2025).

The possibilities of combination therapy using Pt encapsulated by NPs as delivery systems are presented in Figure 7.

#### 4.1.6. Bioorthogonal Reactions Catalyzed by PtNPs

**Figure 8:**
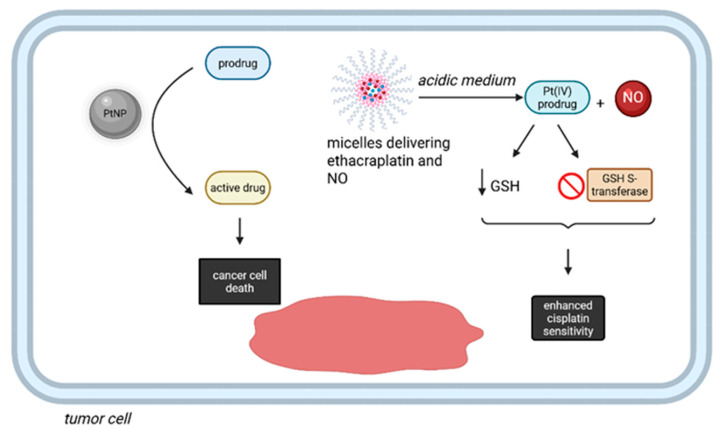
Bioortoghonal reactions performed by Pt compounds in cancer therapy. PtNPs can act within the complex intracellular microenvironment of cancer cells, with in situ activation of anticancer prodrugs into active molecules. Additionally, micelles delivering ethacraplatin and NO can reduce the intracellular GSH and block GSH S-transferase, thus enhancing the sensitivity towards cisplatin. (Created with BioRender https://BioRender.com/52dh5hc, accessed on 1 august 2025).

Pt complexes, in addition to their classical role as cytotoxics, were recently proved to perform bioorthogonal reactions in living organisms [76,77]. Even though some methods based on this approach do not directly employ Pt-based prodrugs, PtNPs serve as catalytic centers for a variety of reactions [76]. For instance, the synthesis of catalytic nanoreactors, represented by PEGylated Pt NPs with special properties, such as a dendritic structure and surface shielding by Pt-S-bonded PEG allow the compound to act within the complex intracellular microenvironment of cancer cells, thus enhancing the in situ biorthogonal activation of anticancer prodrugs [76]. Additionally, a combination between biorthogonal chemistry and an inhalation technique was employed to reverse cisplatin resistance in nonsmall cell lung carcinoma (NSCLC). The effect was achieved by co-delivery of ethacraplatin (i.e., a Pt(IV) prodrug) and nitric oxide (NO) by micelles, specifically targeting cancer cells after inhalation [77]. After arriving in the acidic tumor microenvironment, the Pt(IV) prodrug is released, inhibits GSH S-transferase and reduces the concentration of GSH, leading to increased sensitivity to cisplatin, with additional advantages associated with the release of NO (Figure 8) [77].

A previous study reported the formation of self-assembled coordinative NP BDCNs, which deliver both a Pt(IV) prodrug and a NO prodrug and that accumulate in the tumor. After reduction in the Pt(IV) prodrug to the active Pt(II), activation of NO prodrug occurs by a depropargylation reaction, leading to the release of NO, with synergistic effects [78]. The results of this study show an enhanced the efficiency of bioorthogonal reactions by overcoming the problems posed by the separate administration of the Pt(IV) compound and NO prodrug, such as pharmacokinetic issues [78] (Table 8).

#### 4.1.7. Increased Lipophilicity

Novel nanosystems with enhanced hydrophobicity and passive diffusion capacity, increasing cancer cell accumulation, while combating the drawbacks of conventional therapy and improving the simultaneous chemo-/radiotherapy are emerging. In their development the following objectives are pursued: the deliverance of Pt-based drugs and prodrugs, the pass through the blood–brain barrier, and specifically, the capacity to target malignant cells [79].

It was proved that Pt(IV) prodrugs with increased tumor selectivity formed using biotin and naproxen or stearate in axial position had enhanced anticancer effects. These improved anticancer properties were due to lipophilicity rather than the expression of biotin receptors [80].

Analyzing the importance of lipophilicity of Pt(IV)-based compounds, in an in vitro study, it was suggested that for the most hydrophobic compound tested (namely, diamminedichloridodioctanoatoplatinum(IV)) the formation of nanoaggregates resulted in higher cellular uptake by both passive diffusion and endocytosis than by passive diffusion alone [81]. However, since this compound is active at nanomolar concentrations at which the aggregation in culture media is almost inexistent, this phenomenon should not significantly impact its antiproliferative activity [81]. Another study studied a type of polymeric NP, namely [Pt(DACH)(OAc)(OPal)(ox)] incorporated PLGA NPs, delivering a lypophilic Pt(IV) complex, that showed increased anticancer properties even against cisplatin-resistant cells and reduced systemic toxicity [82] (Table 9).

**Figure 9:**
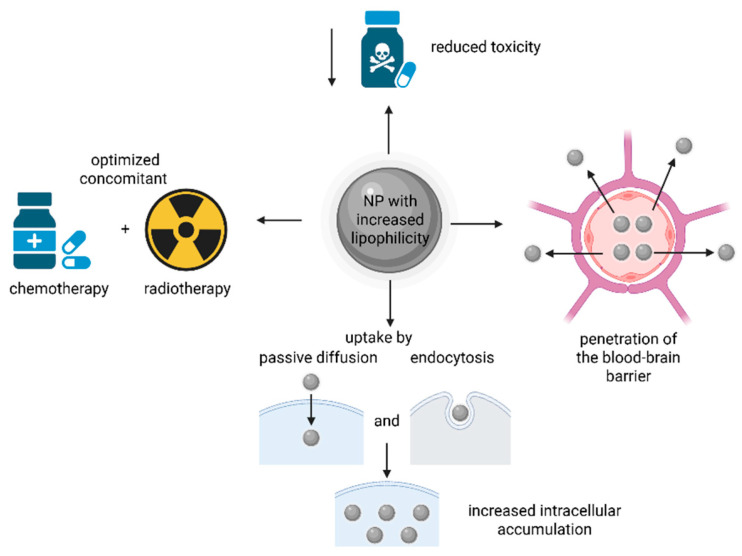
The advantages of NPs with increased lipophilicity in delivering Pt drugs/prodrugs. The increased lipophilicity can help in the penetration of the blood–brain barrier, and the obtained NPs can reduce the systemic toxicity associated with chemotherapy, optimize the chemo-/radiotherapy and increase cancer cell uptake by both passive diffusion and endocytosis. (Created with BioRender https://BioRender.com/efub7kc, accessed on 1 august 2025).

The advantages of nanoformulations with increased lipophilicity are presented in Figure 9.

#### 4.1.8. Targeting Both Malignant and Non-Malignant Cells

One more promising efficient cancer therapy in comparison with the conventional approaches is to target both tumoral and non-tumoral cells at the same time. For this purpose, NP-based codelivery of a Pt(IV) prodrug and Sotuletininb (BLZ-945) showed, after irradiation with light having a 660 nm wavelength, a contraction to small Pt(IV) prodrug-containing NPs and drug deliverance deep inside the tumor, while releasing BLZ-945 around the tumor-associated blood vessels, targeting and damaging tumor-associated macrophages [83].

Even though novel approaches were created for cancer therapy by developing various nanoformulation-based drug delivery systems, most of the studies are limited only to in vivo and in vitro research and the number of approved nanodrugs did not significantly increase throughout the years [84,85]. There are also some drawbacks of the usage of NP-based treatments, such as associated-immunotoxicity, long term toxicity, neural side-effects and costly synthesis [84,86] (Table 10).

### 4.2. Materials Used in Producing NPs for a More Efficient Cancer Treatment

#### 4.2.1. siRNA Technology

The siRNA technology also appears to play a crucial role in regulating Pt-drug sensitivity.

For instance, epigallocatechin gallate-based NPs carrying both Pt(IV) prodrugs and anti-casein kinase 2 alpha 1 (CSNK2A1) siRNA exerted many beneficial effects by extending the circulation time for Pt-based drugs, increasing the accumulation of the drug within the cancerous cells, decreasing the Pt-drug’s renal side-effects and targeting CSNK2A1, by all of these increasing the tumor’s sensitivity to cisplatin [13].

Another study underlined the potential of utilizing the nuclear-targeting lipid Pt(IV) prodrug with amphiphilic and nuclear-targeting properties to increase Pt-DNA cross-linking formation and using siXkr8 (i.e., small interfering RNA targeting XK-related protein 8) to decrease immunosuppression. This RNA would downregulate the exposed phosphatidylserine (i.e., a phospholipid expressed on cancer cell membrane after Pt-based therapy that binds to corresponding receptors on immune cells, leading to lower antitumoral immunity), leading to improved chemoimmunotherapy. The new compound can thereby amplify the anticancer effects of conventional Pt-based drugs and suppress cancer recurrence [87]. Previous studies showed that self-assembled lipid NP LNP co-delivering XPF-targeted siRNA together with a cisplatin prodrug, can enhance cisplatin-associated cytotoxicity by silencing this nucleotide excision repair (NER)-related gene [88]. Additionally, self-assembled PLGA-PEG/G0-C14 NPs, co-delivering REV1/REV3L-specific siRNA and a cisplatin prodrug, can reverse cisplatin resistance by suppressing the genes involved in the error-prone translesion DNA synthesis [89] (Table 11).

**Figure 10:**
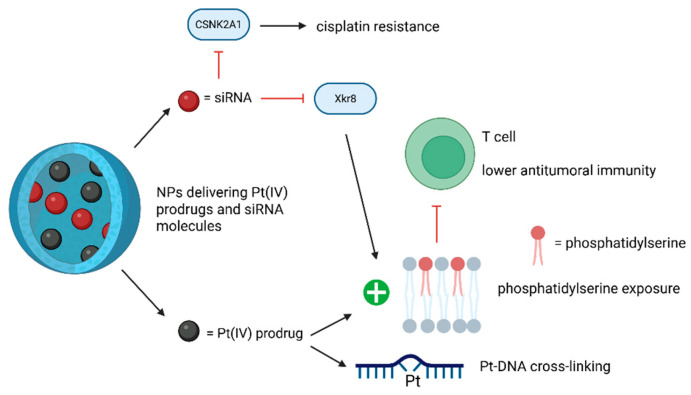
The effects of Pt drugs-siRNA NP-based codelivery systems. The use of siRNAs can reverse cisplatin resistance and reduce treatment-associated immunosuppression. (Created with BioRender https://BioRender.com/x1edlli, accessed on 1 August 2025).

The roles of siRNA technology in the NP-based drug delivery systems of Pt compounds are depicted in Figure 10.

#### 4.2.2. Human Serum Albumin-Based NPs

**Figure 11:**
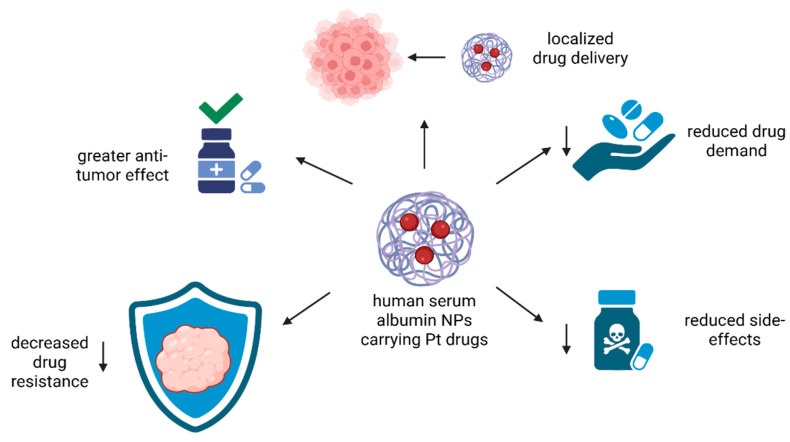
The advantages of Pt complexes delivered by human serum albumin NPs. These NPs can help in localized drug delivery, reduce drug demand, lower the intensity of side-effects, reverse resistance to cisplatin and improve the overall anti-tumor effect. (Created with BioRender https://BioRender.com/yolk07y, accessed on 1 august 2025).

In addition to the results stated above, precise drug delivery with reduced drug demand and lower side-effects could be achieved using human serum albumin (Figure 11).

Human serum albumin was chosen because studies proved that utilizing this protein is effective for active tumor targeting [90]. The reduction-responsive human serum albumin NPs conjugated with OXA demonstrated improved cytotoxicity and lower rates of resistance compared to the free drug utilization in the treatment of triple-negative breast cancer [90]. The same outcomes were reported for AbPlatin(IV) NPs, using human serum albumin as carrier for a lipophilic Pt(IV) prodrug, whose mechanisms of action were analyzed through multi-omics analysis. Modifications of the malignant cell membrane by alterations of glycerophospholipids and sphingolipids and modifications of purine metabolism were discovered [91]. Another study developed human serum albumin–Pt compound complex NPs, HSA-His242-Pt-Dp44mT NPs, which tend to specifically accumulate within cancer cells through the binding of human serum albumin to the secreted protein acidic and are rich in cysteine (SPARC) protein, highly expressed by cancer cells [92]. As a result of this specific binding, enhanced toxicity towards cancer cells and lower side-effects occur [92] (Table 12).

## 5. Conclusions

NP-based drug delivery systems represent a promising approach in overcoming the disadvantages of conventional Pt-based cancer therapy, namely chemoresistance and severe side-effects, that make the drug’s long-term administration difficult. Passive and active targeting cancer cells through specific drug delivery systems could lead to lowered drug demand and toxicity.

They use different strategies to achieve an improved anticancer effect, such as mitochondria targeting, increased stability in the circulation, increased anti-tumoral immunity, multistimuli-responsivity, combination chemotherapy, catalyzed bioorthogonal reactions, increased lipophilicity and the capacity to target both malignant and non-malignant cells. In addition, other materials or techniques used in combination with these NPs can improve their efficacy, such as the use of siRNAs, laser irradiation, deeply penetrating ultrasound radiation, and human serum albumin-based NPs. Due to their numerous mechanisms of action, NP-based drug delivery systems could enlarge therapeutic utilization for multiple cancers. Nevertheless, there are some challenges regarding the use of different types of NPs in the clinic, such as problems linked to biocompatibility, scalability, and regulatory approval.

Several future directions emerge for this type of nanomedicine. Further studies are still needed to better understand the molecular mechanisms of NPs inside different malignant cells in different patients. Additionally, for wide clinical administration, more studies are required to better analyze toxicity and pharmacokinetics, along with physical–chemical standardization.

## Figures and Tables

**Table 1:** The side-effects induced by cisplatin therapy.

Cisplatin-Induced Side-Effects	Complications and Symptoms	Mechanisms
Renal Toxicity	acute kidney injury	pyroptosis
	hypomagnesemia	oxidative damage
	Fanconi-like syndrome	inflammation [12]
	distal renal tubular acidosis	
	hypocalcemia	
	wasting renal salt	
	hyperuricemia [13]	
Allergic Reactions	skin rash	type I allergic reactions [14]
	flushing abdominal cramping itchy palms back pain [14]	
Gastrointestinal Disorders	colitis	direct epithelial damage
	diarrhea [15]	inflammation
		disruption of the normal integrity of the gastrointestinal mucosa
		microbiome alteration [16]
Hemorrhage	reduced chemotherapy dosages	apoptosis through the ERK signaling pathway [17,18]
	postponed treatment	
	bleeding	
	unfavorable oncological outcomes [17,18]	
Hearing Loss	tinnitus	direct damage to mitochondrial and nuclear DNA
	high-frequency hearing loss	apoptosis caused by cell cycle arrest and activation of p53
	decreased ability to hear normal conversation [19]	generation of ROS by the activation of NADPH oxidase 3 and xanthine oxidase [19]

**Table 2:** Nanoparticle properties and functional mechanisms for platinum drug delivery.

Nanoparticle properties and functional mechanisms for platinum drug delivery	mithocondria targeting
	increased stability in the circulation
	increased anti-tumoral immunity
	multistimuli-responsive drug delivery systems
	siRNA technology
	nanozyme-based photocatalytic conversion of Pt(IV) to Pt(II)
	combination chemotherapy
	deeply penetrating ultrasound radiation activation
	bioorthogonal reactions catalyzed by PtNPs
	increased lipophilicity
	human serum albumin-based NPs
	targeting both malignant and non-malignant cells

**Table 3:** Latest Pt nanoparticle-mediated drug delivery systems and formulation targeting mitochondria.

Compound	Type of NP	Components	Characteristics	Main Finding	References
LND-S-S-Pt-TPP/HA-CD	Self-assembled nanotargeted drug delivery system	HA-CD TPP LND cisplatin prodrug (Pt(IV))	HA-CD targets membrane CD44	Synergistic destruction of cis-platin-resistant lung cancer cells by disruption of mitochondria	[33]
			TPP targets mitochondria		
			LND damages mitochondria and inhibits glycolysis		
			GSH-mediated reduction in Pt(IV) with mtDNA damage		
PL-III	Self-assembled, spherical shaped NPs	Oxoplatin Lithocholic acid Heptanoate	Halting cell cycle in the S and G2 phases	10-fold higher cytotoxicity compared to cisplatin in PC3 cells	[34]
			Damaging DNA		
			Disruption of mitochondrial membrane potential		
			Increased ROS production	Mechanisms of action requiring reduction in the Pt(IV) core to Pt(II) and simultaneous release of lithocholic acid, with unique cytotoxicity	
			Alteration of mitochondrial bioenergetics		
			Upregulation of pro-apoptotic proteins and reduction in anti-apoptotic ones from BCl-2 family		
TPP-Pt	Ultrasmall peptide-coated platinum nanoparticles	TPP peptides Ultrasmall PtNPs	Monodispersity	Deliverance of TPP-Pt to the thermally susceptible tumor mitochondria, with minimal side-effects	[35]
			High stability		
			Biosafety		
			Enhanced uptake of cancer cells		
			Priority of mitochondria		
NTSB	Pt-based nanoprojectile	DSPE-PEG_2K_-IR780 TSB (FFa-Pt(IV) prodrug-TPP)	DSPE-PEG_2K_-IR780 enhances cellular internalization and equips the carrier with PDT and PTT capabilities	Enhanced sensitivity of tumor cells to Pt-based chemotherapeutic drugs	[36]
			TSB targets cellular mitochondria releasing (OXA) (which attacks the mtDNA) and FFa (which disrupts the electron transport chain)		

**Table 4:** Latest Pt nanoparticle-mediated drug delivery systems and formulation increasing blood stability.

Compound	Type of NP	Components	Characteristics	Main Finding	References
PEG-OXA NPs	Micellar NPs	OXA PEG long lipid chains	Optimized absorption, delivery and elimination	Facilitation of rapid bioactive OXA release in tumor cells Manifestation of high stability in blood in vitro Increased half-life in vivo	[9]
			Increased antitumor efficacy		
			Decreased side-effects		
			Enhanced inhibition of growing tumors Stability in whole blood or plasma due to the low critical micelle concentration		
ERY1-PEG_3k_/PPA-Pt(IV)-NCs	Nanocrystals	A small-molecule Pt(IV) prodrug [Pt(DACH)(PPA)(OCOCH_2_CH_2_COOH)(ox)] dielectric nanocrystals PEG_3k_ ERY1	Enhanced stability due to the ERY1-mediated binding to erythrocytes in the bloodstream	Overcoming of shortcomings (i.e., reduced circulation, failure to accumulate in the tumor, and dose-limiting toxicity) of traditional Pt-based chemotherapy through an erythrocyte-delivered and NIR photoactivatable Pt(IV) nanoprodrug	[37]
			Prolonged circulation in the blood		
			Minimized side-effects		
			Increased Pt accumulation in tumor through erythrocyte delivery approach		
			Enhanced immunopotentiation		
			Oxaliplatin release in a controllable manner upon irradiation		
PtNPs with protein coronas	Self-assembled PtNPs with protein coronas	Cisplatin HAS and other blood proteins	Rapidly generated in vivo in human blood upon treatment with cisplatin	Functioning as a biocompatible drug delivery platform for chemotherapy-resistant tumor treatment	[38]
			Accumulation in tumors		
			Persistence in the body for an extended period of time by NPs’ interaction with HSA coating them		
			Consumption of intracellular GSH		
			Activation of apoptosis		
			Capacity to reverse tumor resistance to daunorubicin		
Cisplatin-Tetrac-His-P(AEMA-co-PEGDMA)	Nano-hydrogel	Aminoethyl methacrylamide PEG dimethacrylate L-histidine Cisplatin tetraiodothyroacetic acid	Suitable nano size	Synthesis of a novel nano-hydrogel (NH) and depicted its application in the process of targeted delivery of cisplatin	[39]
			Relatively good hemo-compatibility		
			pH-responsive drug release pattern		
			Active targeting via integrin receptors		
			Improved pharmacokinetic parameters		
Cisplatin-EDTA-MMSN @ HA	MSN	Magnetic mesoporous silica nanoparticles (MMSNs) EDTA Hyaluronic acid cisplatin	pH-responsive behavior	Synthesis of a novel multifunctional pH-responsive, biocompatible and biodegradable nanoplatform for efficient drug delivery and magnetic resonance imaging	[40]
			Improved internalization in cancer cells overexpressing CD44 receptor compared to normal cells		
			Improved pharmacokinetic parameters		
			High adsorption capacity of cisplatin		
			Reduced side-effects		
			Theranostic nature		

**Table 5:** Latest Pt nanoparticle-mediated drug delivery systems and formulations increasing anti-tumoral immunity.

Compound	Type of NP	Components	Characteristics	Main Finding	References
NP(3S)s	Reduction-responsive NPs formed though self-assembly	Polymers containing trisulfide bonds, using bis(2-hydroxyethyl) trisulfide, 1,2,4,5-cyclohexanetetracarboxylic dianhydride and mPEG-5k CisPt(IV) prodrug	DNA damage	NP(3S)s holding great promise for clinical translation due to low toxicity profile and potent anticancer activity, by enhancing antitumor immunity and OS pathways	[41]
			Activation of STING pathway		
			Activation of T cells		
			Activation of OS mechanisms		
			Reduction to and release of Pt(II) and H_2_S by GSH		
NP-Pt-IDOi	Self-assembled polymeric NPs	PHPM Pt(IV)-C12 NLG919	DNA damage	Superior anticancer activity in vitro and in vivo in mouse models of osteosarcoma Efficient combination of chemotherapy and immunotherapy	[42]
			Induction of STING pathway		
			Increased anti-tumor immunity		
			Activation of cytotoxic CD8+ T cells by tryptophan metabolism inhibition		
ALN-OXA NPs	Self-assembled lipid NPs	Lipo-OXA-ALN	Targeting osteosarcoma cells	T enhancing the therapeutic effects against osteosarcoma by osteosarcoma targeting capacity, increasing chemotherapy sensitivity and improving the immune microenvironment.	[43]
			Enhanced 189 intracellular uptake of OXA		
			Inhibition of cancer cell activity		
			Increased antitumoral immunity		
			Increased M1/M2 macro-phage ratio		
			Modifying the tumoral microenvironment		
NP3	Self-assembled NPs	P1 polymer obtained from DSB, 1,2,4,5-Cyclohexanetetracarboxylic Dianhydride and mPEG_5k_-OH Pt(IV)-C16 IR1061	Increasing ICD in tumors after irradiation with NIR-II	Increased therapy results for triple-negative breast cancer 4T1 compared to either OXA or NIR-II-based photothermal therapies alone	[44]
			Increased Pt-DNA binding		
			Higher DNA damage and apoptosis		
IONP@BCP@[PtCl(GUDCA)en] NPs	Pt compound conjugated with iron oxide NPs	IONPs BCP [PtCl(GUDCA)en]	Enhanced Pt-associated cytotoxicity	Cisplatin-derived agents together with high value of IONPs as drug delivery systems and immunogenic cell death	[45]
			Activation of endoplasmic reticulum stress pathways with activation of ICD		
			Biocompatibility in biological systems		
I-Pt NPs	Hyperthermia sensitive Pt NPs	BMS-1 Mal-modified PEG PtNPs	Improved biocompatibility	Synergistic augmentation of immunological responses and photothermal ablation of tumors Prevention of cancer relapses and metastasis	[46]
			NIR laser irradiation-mediated photothermal conversion and PTT mediated tumor ablation, with BMS-1 release and Mal exposure		
			Capture of tumor-associated antigens by exposed Mal and their presentation to APCs		
			BMS-1-mediated inhibition of immunosuppression and stimulation of immune response		
OxPt/BP	Nanoscale coordination polymer	OXA 2-bromopalmitic acid	Inhibition of palmitoyl acyltransferase DHHC3 Downregulation of PD-L1 expression in both cancer cells and dendritic cells	Potential of NCPs to simultaneously reprogram cancer cells and DCs for potent cancer treatment	[47]
			Enhanced DC maturation		
			Increased intracellular OS		
			Enhanced cancer ICD		
			Stimulation of infiltration and activation of cytotoxic T lymphocytes		
			Reduction in the population of immunosuppressive regulatory T cells		

**Table 6:** Latest Pt nanoparticle multistimuli-responsive drug delivery systems.

Compound	Type of NP	Components	Characteristics	Main Finding	References
BT-Pt (IV)@PEG/CaCO_3_	Liposomes	CaCO_3_ NPs BT OXA-DSPE DSPE-PEG Cholesterol DPPC	High Stability	Biocompatible and reliable substrate for establishing pH-mediated drug delivery methods Promising for possible therapeutic application	[50]
			Rapid pH-mediated degradation		
			Increased OS in cancer cells		
			Mitochondrial and DNA damage inside cancer cells		
UCNP/Pt(IV)-RGD	Upconversion NPs	NaYF_4_:Yb,Tm@NaYF_4_ UCNPs DSPE-PEG_2000_ PHB-Pt(IV)-18Cprodrug c(RGDyK)	High biocompatibility	Multifunctionality Precision through enhanced therapeutic efficacy, targeted delivery, multimodal imaging Personalized treatment	[48]
			Tumor specificity		
			Profound cytotoxicity upon UCL irradiation and GSH reduction		
			Real-time UCL imaging capacity		
DDNPs	Dual-Sensitive Dual-Prodrug Nanoparticles	Pt(IV) prodrug DMC nanoplatform	Photosensitivity, with generation of active Pt(II) from inert Pt(IV) under UVA light	Powerful synergistic anticancer effect in vitro and in vivo, with great potential as a safe and multifunctional drug delivery system for precise nanomedicine in clinical treatments	[51]
			Acid-sensitivity, with release of DMC and blockage of the DNA repair pathway		
			Endo/lysosomal escape for better photoactivated chemotherapy		
DDPoly NPs	Self-assembled dual-drug polymer micellar NPs	DMC MPEG	Acid- and reduction-sensitivity	Enhanced anticancer efficacy against cancer cells compared to SDPoly NPs, highlighting its potential for nanomedicine development	[52]
			Tumor-specific activation		
			Blocking DNA repair, with enhancing Pt(II)-induced apoptosis		
NPS-G-Fe	Metallo-nano prodrug	Polyaspartamide-PEG Chlorin e6-Pt(IV) Fe^3+^ gallic acid PEG-CS	Activation by both acidic tumor microenvironment and light, resulting in the activation of both chemotherapy and PDT	A versatile platform for the codelivery of therapeutic agents, exhibiting significant potential for synergistic tumor therapy while minimizing adverse side-effects	[53]
			Metallo-triggered ferroptosis		
			Polarization of TAM		
Pt(IV)-UA NPs	Self-assembly of dual prodrug amphiphile into NPs	Pt(IV)-UA-PEG dual prodrug amphiphile	High circulation time	Development of a stimuli-responsive dual prodrug amphiphile nano-assembly providing a new strategy to overcome drug resistance	[58]
			High tumor accumulation		
			High antitumor activity		
			Lack of side-effects associated with conventional therapy		
			Reversing cisplatin resistance		
			Drug release in intracellular reductive and acidic environments		
Pt-Coordinated Dual-Responsive Nanogels	Nanogel	HA-βCD PEI cisplatin	Hyaluronidase and GSH responsiveness, releasing the loaded drugs	This dual-responsive nanogel-based platform can serve as a multifunctional platform capable of specific delivery of desired drugs to treat cancer or other diseases	[59]
			Small-molecule drug and protein loading and intracellular delivery capacity		
			Capacity to co-deliver different cargoes to realize combination cancer therapy		
FMN@TACN AuNPs	Supramolecular nanozyme	AuNPs C11-thiol bearing a 1,4,7-triazacyclononane headgroup FMN	Photocatalyzation of the reductive activation of the prodrug cis,cis,trans-[Pt(NH_3_)_2_(Cl_2_)(O_2_CCH_2_CH_2_COOH)_2_] to cisplatin in the presence of an electron donor through an excited-state electron transfer process	TACN AuNPs are suitable components to develop supramolecular nanomaterials capable to carry out flavin-mediated catalytic reactions using Pt(IV) prodrugs as substrates	[54]
			Potential strategy to control spatio-temporally the effects of Pt anticancer drugs via light activation and catalytic amplification		
NC1–NC4	Micellar NPs	Photosensitive Pt(IV)–azide prodrug complexes based on cisplatin and oxaliplatin (C1-C4)	Rapid release of biologically active Pt(II) and enhanced cytotoxicity upon UVA irradiation	Pt(IV) complexes, specifically when formulated into micellar nanoparticles have potential to offer a robust platform for controlled delivery and selective activation of Pt-based anticancer therapeutics	[55]
			Great stability in the dark		
			Increased uptake by cancer cells		
			Enhanced circulation time in the bloodstream		
			Decreased systemic toxicity		
			Increased tumor growth inhibition		
NP^s^	Self-assembled NPs	Pt1 (Pt(IV) prodrug) Hemoglobin DSPE-PEG_2K_	Biocompatibility and sonosensitivity via hemoglobin	The first example of Pt(IV) prodrug NP activation upon exposure to ultrasound radiation for deep tissue penetrating anticancer therapy	[56]
			Activation upon deeply penetrating ultrasound radiation with tumor eradication via apoptosis		
			Treatment of deep-seated or large tumors		
			Selective accumulation inside the tumor		
			Stability under physiological conditions		
P-Rf/cisPt(IV) NPs	Self-assembled NPs	mPEG-b-PLG Ac-cisPt(IV)-OH Rf-(OH)_2_ mPEG-NH_2_	Enhanced activation upon ultrasound radiation via riboflavin, mediated by the superoxide ions	Enhancing activation efficacy of Pt(IV) prodrugs under low-intensity ultrasound conditions Innovative ultrasonic chemical reaction mechanism Novel insights into ultrasound-mediated activation of Pt(IV) prodrugs	[57]
			Significant antitumor effects even under low-intensity ultrasound radiation		

**Table 7:** Latest achievements in the production of NPs for combination chemotherapy.

Compound	Type of NP	Components	Characteristics	Main Finding	References
PFS-NP	Self-assembled metal–phenolic network NPs	A disulfide bond-based amphiphilic polyphenol A dopamine-modified cisplatin prodrug Fe^3+^ ions	GSH-responsiveness	Innovative cisplatin prodrug NP approach offering a promising reference for minimizing side-effects and optimizing the therapeutic effects of cisplatin-based drugs, for synergistic chemo-immunotherapy	[4]
			Consumption of intracellular GSH with disruption of the redox homeostasis		
			Amplification of the OS		
			Generation of ICD, with subsequent activation of the anti-tumor immune system		
iAIO@NSe-Pt	Amorphous ferric oxide-coating selenium core–shell NPs	Ferric oxide shell AIO on the surface Pt(IV) prodrug Se core	Avoidance of the inactivation of the Pt(IV) prodrug in the blood and increasing its accumulation in the tumor	Excellent tumor targeting, biocompatibility and anti-tumor efficiency in vitro and in vivo, and proving to be a novel example of a self-preservation Pt(IV) nanoplatform for H_2_O_2_ depletion-mediated tumor anti-angiogenesis, apoptosis, and ferroptosis	[61]
			Leading to cellular H_2_O_2_ deficiency and cancer cell ferroptosis		
			GSH consumption, with increased OS		
			Effective apoptotic cell death		
			Inactivation of SOD1, with increased OS		
			By decreasing H_2_O_2_, causing reduction in VEGF-A expression, blocking tumor angiogenesis, disruption of mitochondrial respiration and cancer angiogenesis		
Pt&Fe_3_O_4_@PP	polypeptide NPs	c,c,t-[Pt(NH_3_)_2_Cl_2_(O_2_CCH_2_CH_2_CO_2_H)_2_] prodrug PGA PLL PEG-NHS	Theranostic agents for combination therapy guided by T2-weighted MRI	New strategy to construct polypeptide-based theranostics with tumor-microenvironment-activatable cascade reactions, promising for cancer treatment application	[62]
			Pt-based drug and Fe^2+/3+^ release triggered by the reducing reagent and pH conditions		
			Ferroptosis induction upon entering tumor microenvironment		
			Induction of apoptosis in tumor cell		
			Efficient inhibition of cancer cell growth		
			No significant systemic toxicity		
PTCG NPs	Metal–polyphenol-coordinated NPs	EGCG phenolic Pt(IV) prodrug PEG-*b*-PPOH	High stability in blood circulation	A promising strategy to develop advanced nanomedicine for cascade cancer therapy by efficiently combining chemotherapy and chemodynamic therapy with excellent anticancer efficacy	[63]
			Strong metal–polyphenol coordination interactions		
			Efficient drug release after cellular internalization		
			Increased OS by H_2_O_2_ generated by cisplatin and Fe-based Fenton reactions		
			Avoidance of systemic toxicity		
Pt-Fe NCPs	Nanostructured coordination polymers	Pt(IV) prodrug bearing bis-catechol groups Fe(III) ions	Dual pH- and redox-sensitivity	Opening a future path for investigation of intranasal Pt nanoderivatives for brain tumor treatment, overcoming the blood–brain barrier permeability challenges, even in high-grade brain tumors such as glioblastom.	[64]
			Controlled release Cytotoxic effect comparable to cisplatin		
			Slower release profile and activation period for the Pt(IV) prodrug activation		
			Increased accumulation of Pt in tumors in vivo		
			Complete cure and prolonged survival of the tested cohort in vivo		
			Intranasal administration		
			Reduced side-effects		
Cetuximab-Pt-INPs	Ultrasonic emulsification-based self-assembled NPs	MPEG-PLA-Pt(IV) prodrug (condensation between c,c,t-[Pt(NH_3_)_2_Cl_2_(OOCCH_2_CH_2_COOH)(OH)] and MPEG-PLA) Mal-PEG-PLA Indocyanine green cetuximab	Specific delivery of drugs to EGFR-	A novel kind of cetuximab-decorated and NIR-activated NPs that can be selectively internalized into cancer cells via receptor-mediated endocytosis Promising potential for targeted and effective therapy against EGFR-hyperexpressing cells of epidermoid carcinoma	[65]
			Hyperexpressing cancer cells through cetuximab		
			Higher cytotoxicity and cancer cell uptake upon NIR irradiation		
NPTG	PLGA-PEG-based NPs	Oxaliplatin(IV)(Gem)_2_ (PTG) PLGA-PEG-COOH	Reduced side-effects	Synergistic combination of established chemotherapeutic agents within a Pt(IV) scaffold, coupled with the potential benefits of NP delivery systems for more effective and tolerable anticancer treatments	[66]
			Overcoming drug-resistance mechanisms		
			Enhanced cancer targeting		
			Increased growth inhibition		
			Release of active molecules -Pt(II) and gemcitabine-under tumor-associated reductive medium		
^SS^CV_5_ NPs	Nanoprecipitation-based self-assembled NPs	Pt-VES prodrug PSSV polymer	GSH scavenging	A safe and facile nano-sensitizer and a promising route for the neoadjuvant chemoradiotherapy of cervical cancers.	[20]
			mitochondrial damage		
			reverse of the cisplatin resistance by consuming intracellular GSH		
			DNA damage and apoptosis		
			Sensitization of cancer cells to X-ray radiation		
			Accumulation inside and growth inhibition of cisplatin-resistant cancer cells		
			Reduced side-effects		
			High Pt(IV) prodrug loading capacity		
IR780@Pt NPs	Supramolecular self-assembly-formed NPs	Pt-CD prodrug Ad-BH IR780	NIR-irradiation induced mitochondrial dysfunction of cancer cells	Innovative nanomedicine IR780@Pt NPs mediating targeted induction of mitochondrial dysfunction to potentiate chemotherapy High significance of combinatorial therapy by multifunctional nanotheranostics for synergistically enhanced cancer therapy	[67]
			Downregulation of key proteins of the NER pathway, with enhanced chemotherapeutic effect		
			NIR fluorescence and photoacoustic imaging capacity		
			Increased tumor inhibition performance		
PVPt@Cy NPs	Hydrophobic interaction-induced self-assembled NPs	c,c,t-[Pt(NH_3_)_2_Cl_2_(OH)(O_2_CCH_2_CH_2_CH_2_CO_2_H)] α-tochoferol mPEG_2K_-OH PMA Cy	Synchronous chemotherapy, PDT and PTT	Promising approach in imaging-guided combined chemo-phototherapy	[68]
			disaggregation under acidic, reductive conditions and NIR irradiation, with photothermal conversion and OS generation		
			An enhanced in vitro anticancer efficiency under irradiation		
			Strong NIR fluorescence and photothermal imaging		
Pt–I–IR780 NPs	Self-assembled NPs	P1 polymer (Pt(IV) in the main chain and pendant pair-wise iodides) IR780	NIR irradiation-mediated mild hyperthermia	External NIR light to irradiate NPs with photothermal sensitizers to produce mild hyperthermia, which increases the formation of Pt-DNA interstrand cross-links with efficient Pt-based chemotherapy	[69]
			Increased GSH-mediated reduction in Pt(IV) to Pt(II)		
			Reduced GSH-mediated detoxification of cisplatin		
			Increased Pt-DNA interstrand cross-links		
ABC triblock Pt-prodrug NPs	Self-assembled NPs	PBI-tagged ABC-triblock PCL Cisplatin PEG	Excellent shielding against cisplatin detoxification by the intracellular GSH species	Nanoformulation with both enhanced efficiency against cancer cells and capacity to monitor intracellular administration of non-luminescent cisplatin	[70]
			Extracellular stability and intracellular lysosome-mediated biodegradation of PCL with release of Pt-drug		
			Enhanced cancer cell growth inhibition		
			Simultaneous monitoring and delivery aspects of the Pt-prodrug		
PDDN	Emulsion interfacial polymerization-based NPs	Pt(IV) prodrug derived from OXA mitochondria-targeting cytotoxic peptide	High drug loading efficiency	Appreciable combination efficacy on both cell line-derived and patient-derived xenograft lung cancer model	[71]
			Combination therapy		
			Precise drug ratio		
			Ph-induced drug release		
			Activation upon GSH-mediated reduction		
			High biocompatibility and reduced side-effects		
			Inhibition of drug efflux and DNA damage repair		
			Appreciable antitumor effects		
NP(Se)s	Micellar NPs	mPEG_5000_-(CHTA-*co*-DSB)-mPEG_5000_ (diselenium bond containing polymer) phospholipid like morpholinized Pt(IV) prodrug (C16-CisPt-TA)	GSH depletion and ROS generation	A promising strategy to break the redox balance for maximizing the efficacy of Pt-based cancer therapy	[72]
			Significant antitumor effect		
			Biocompatibility		
			Decreased drug resistance		
NP-Pt-USP1i	Self-assembled NPs	PHHM C527 (USP1i) Pt (IV)–C12 prodrug	GSH sensitivity	USP1i combined with a Pt(IV) prodrug in NPs could inhibit the growth of liver cancer, with the possibility of a future precise cancer therapy with cisplatin and USP1i to overcome cisplatin resistance in the clinic	[31]
			Inhibition of the DNA damage repair		
			Increased sensitivity of tumor cells to cisplatin		
			Enhanced anticancer effect		
PEG-Pt(IV)@DOX NPs	Co-self-assembled NPs	PEG-Pt(IV) prodrug doxorubicin	Longer blood retention	A promising platform for combination chemotherapy of hypoxic solid tumors in the clinic	[73]
			Enhanced accumulation in tumor sites		
			Light irradiation-mediated in situ Generation of O_2_, with release of active Pt(II) and doxorubicin		
			Increased ROS		
			Enhanced cytotoxicity		
			Alleviation of hypoxia-induced MDR of doxorubicin		
			Reduced side-effects		
Nanoplatin^DTR^	Self-assembled NPs	Polyplatin^DT^ caspase-3 cleavable peptide	Delivery of drugs and fluorophores concomitantly at a precise D/T ratio	The first theranostic nanoplatform with anticancer drugs, drug tracers, and drug efficacy reporters that can work in concert to provide insight into the drug fate and mechanism of action	[74]
			Tracking of the Pt drugs via NIR-II imaging		
			Evaluation of the therapeutic efficacy via the apoptosis reporter		
PCPt NPs	Self-assembled NPs	PCPt	Fixed drugs loading ratio	PCPt NPs as a promising platform for Pt and curcumin co-based combination chemotherapy circumventing mono-chemotherapy limitations	[75]
			High drugs loading content		
			Improved solubility and stability of curcumin		
			Drug release under reductive conditions		
			Efficient synergistic chemotherapy		
			Excellent reversal ability of tumor resistance to cisplatin		
			Effective intracellular uptake		
			Enhanced cell proliferation inhibition		

**Table 8:** Current progress in bioorthogonal reactions catalyzed by PtNPs.

Compound	Type of NP	Components	Characteristics	Main Finding	References
PEG-dPt-2	Dendritic NPs	dPt-2 (formed from H_2_PtCl_6_ and ascorbic acid assisted by Pluronic F-127) PEG-SH	Bioorthogonal catalytic nanoreactors to enable the in situ release of anticancer drugs through depropargylation reactions	Developing noble metal-based nanodevices in bioorthogonal catalysis Offering new opportunities to modulate the optical properties and bioactivity of small molecules in the highly crowded intracellular environment	[76]
			High biocompatibility		
			High surface area-to-volume ratio		
			Increased catalytic performance of Pt NPs		
EA-Pt@M_DBCO_	Self-assembled polymeric micelles	EA-Pt PEOz-*b*-PLA-GSNO DSPE-PEG_2000_-DBCO	Improved targeting performance toward pulmonary cancerous regions after prelabeling with azide via inhalation	Inhalable EA-Pt@M_DBCO_ effectively reversed cisplatin resistance in an NSCLC model New therapeutic option for lethal NSCLC in clinic	[77]
			pH-sensitivity		
			Depletion of intracellular GSH		
			Inhibition of GSH-S-transferase activity		
			Improved therapeutic outcome against NSCLC		
			Reversion of cisplatin resistance		
			NO release within GSH-enriched cancer cells, with synergistic effects		
			Increase in the survival rate		
			In vivo biosafety		
BDCNs	Self-assembled coordinative NPs	Compound A—Pt(IV) prodrug bearing two terminal carboxyl groups Compound B—NO prodrug *O*^2^-propargyl diazeniumdiolate with two terminal carboxyl groups Fe^3+^ ions	Accumulation in tumor through passive targeting	Overcoming of current bio-orthogonal chemistry shortcomings, especially the separated administration and targeting ability	[78]
			Stability in the circulation		
			Reduction in Pt(IV) to cisplatin in cancer cells		
			Cisplatin-mediated depropargylation of prodrug B to generate high levels of NO		
			Flexibly adjusted as needed in proportion to two prodrugs		
			Cascade reactions specifically initiated at the tumor site with both synergistic anticancer activity and reduced side-effects		
			Avoidance of pharmacokinetic complexity of separated administration		
			Enhanced the efficiency of bio-orthogonal reactions		
			Fe^3+^-initiated Fenton reaction-synthesized produced hydroxyl radicals with tumoricidal activity		

**Table 9:** Latest advancements in the production of NPs delivering lipophilic Pt-based compounds.

Compound	Type of NP	Components	Characteristics	Main Finding	References
Dicarboxylate Pt(IV) Prodrugs Self-assembled NPs	Self-assembled NPs	Dicarboxylato Pt(IV) prodrugs- Ace, But, Hex, Oct	Formation of nanoaggregates at the highest concentration tested for Oct and Hex	This study underlined the important link between cancer cell uptake and lipophilicity of the prodrug. Lipophilic compounds can form nanoaggregates, especially at high concentrations, which can improve the cellular uptake through both passive diffusion and endocytosis but do not enhance the antiproliferative effect of complexes active at nanomolar concentrations.	[81]
			Higher cellular uptake by both passive diffusion and endocytosis for Oct		
			Antiproliferative activity not significantly impacted by aggregation for Oct		
			Increased lipophilicity		
			Disaggregation in the complete cell culture media (by virtue of the presence of bovine serum albumin)		
			Low zeta potentials causing low stability and high dispersity		
[Pt(DACH)(OAc)(OPal)(ox)] incorporated PLGA NPs	Pt(IV) prodrug-incorporated polymeric NPs	[Pt(DACH)(OAc)(OPal)(ox)] PLGA lipoid E80 OCA	Unique potency against a panel of cancer cells, including cisplatin-resistant tumor cells	Modification of OXA into a lipophilic Pt(IV) complex containing both lipophilic and hydrophilic axial ligands improves performance and facilitates incorporation into polymeric NPs	[82]
			Enhanced in vitro cellular Pt accumulation, DNA platination, and antiproliferative effect compared to OXA		
			Decreased tumor growth rates compared to control and OXA treatment groups in vivo		
			Reduced systematic toxicity and side-effects by incorporating the prodrug in PLGA NPs.		

**Table 10:** Current directions in targeting both malignant and non-malignant cells.

Compound	Type of NP	Components	Characteristics	Main Finding	References
BLZ@S-NP/Pt	core–shell–corona self-assembled NPs	mPEG_45_-*b*-PHEP_20_/Pt TK-PPE BLZ-945 Ce6	Differentially targeting tumor cells and TAMs	BLZ@S-NP/Pt differentially and precisely delivering agents to TAMs and tumor cells located in different spatial distribution Synergistic anticancer effects in multiple tumor models	[83]
			Shrinkage to small Pt(IV) prodrug-conjugating NPs Deep tumor penetration under light irradiation		
			Release of BLZ-945 in the perivascular regions of tumor to deplete TAMs		
			Inhibition of tumor growth, prevention of metastasis, and increase in survival period under light irradiation		
			Reversion of the immunosuppressive tumor microenvironment and activation of the T cell-mediated antitumor immune response		
			Prolonged circulation in vivo		
aPD-1/CDDP@NPs	Lipid-coated NPs	cis-[Pt(NH_3_)_2_(H_2_O)_2_]_2_(NO_3_)_2_ DOPA DOTAP DSPE-PEG-AA Cholesterol aPD-1	Synergistic immuno-chemotherapy	Development of a microneedle patch loaded with pH-responsive tumor-targeted lipid NPs which allows local delivery of aPD-1 and cisplatin precisely to cancer tissues for cancer therapy	[85]
			Effective increase in the immune response		
			Enhanced tumor regression		
			Microneedle-induced T-cell response		
			Lockage of PD-1 in T-cells by aPD-1, with enhanced T-cell activation		
			Increase in direct cytotoxicity of cisplatin in tumor cells		
			Increased response rate in the animal model unresponsive to aPD-1 systemic therapy		
			Improved tumor targeting		
			Decreased systemic toxicity		

**Table 11:** Latest achievements in the production of NPs co-delivering Pt-based compounds and siRNAs.

Compound	Type of NP	Components	Characteristics	Main Finding	References
Pt-TPNs/siRNA	Tea Polyphenol NPs	EGCG anti-CSNK2A1 siRNA cisplatin(IV) prodrug	Extended circulation time	Pt-TPNs/siRNA not only enhances the anticancer effects but also mitigates cisplatin-induced renal toxicity, achieving efficacy while reducing toxicity	[13]
			Increased accumulation within the cancer cells		
			Decreased Pt-drugs-associated renal side-effects		
			Augmented cisplatin susceptibility of cancer cells		
NPt(IV)@siXkr8	Self-assembled lipid NPs	Lipo-Pt(IV)-R_8_K siXkr8	Reduction in phosphatidylserine level exposure, with subsequent decrease in immunosuppression	Amplification of conventional Pt-based drug anticancer effects of and suppression of cancer recurrence through improved chemoimmunotherapy	[87]
			Increased Pt-DNA cross-linking formation		
			Accumulation in tumors		
			Cancer cell nucleus targeting		
			Activation in a reduced microenvironment		
LNP	Self-assembled lipid NPs	cisplatin prodrug XPF-targeted siRNA	Efficient transport of the molecules into cells	A multi-targeted NP system that can specifically silence an NER-related gene to promote apoptosis induced by cisplatin, especially in cisplatin-refractory tumors	[88]
			DNA damage		
			Downregulation of both mRNA and levels of XPF, potentiating the Pt drug		
			Improved cytotoxicity in both cisplatin-sensitive and -resistant human lung cancer cells		
PLGA-PEG/G0-C14 NPs	Self-assembled NPs	[Pt(NH_3_)_2_Cl_2_(O_2_C(CH_2_)_8_CH_3_)_2_] cisplatin prodrug REV1/REV3L-specific siRNAs PLGA-PEG block copolymers G0-C14 cationic lipid	Suppression of REV1 and REV3L involved in the error-prone translesion DNA synthesis	Co-delivering a DNA-damaging chemotherapeutic and siRNAs that impair the cell’s ability to repair the DNA damage, which can sensitize cancer cells to chemotherapeutics, and shows superior tumor inhibition compared with monochemotherapy	[89]
			Sensitization of resistant tumors to chemotherapy		
			Reduced frequency of acquired drug resistance of relapsed tumors		

**Table 12:** Latest insights in developing human serum albumin-based NPs.

Compound	Type of NP	Components	Characteristics	Main Finding	References
OSHB and OSHG NPs	Desolvation technique-based NPs	OXA-SS-HSA conjugate BAC (for OSHB) GLUT (for OSHG)	Small size, uniform surfaces, and a satisfactory encapsulation coefficient	Release of OXA in the tumor milieu via glutathione-sensitive prodrug degradation and NP disassembly Smart nanomedicine strategy to realize a robust anticancer response with reduced off-target effects in triple-negative cancer therapy	[90]
			Active tumor targeting via HSA		
			Dual reduction sensitivity to GSH (greater for OSHB)		
			Enhanced cytotoxicity and cell death (greater for OSHB)		
			Reduced drug resistance		
			Biocompatibility		
			Excellent tumor-suppressing efficacy		
AbPlatin(IV)	Self-assembled NPs	CisPt(IV) hydrophobic Pt(IV) prodrug HSA	Better tumor-targeting effect	Development of abplatin(IV) and the use of multi-omics for the mechanism elucidation of prodrug Progress in clinical translation of the prodrug	[91]
			Greater tumor inhibition rate even on cisplatin-resistant cells		
			Alterations of glycerophospholipids and sphingolipids in malignant cell membranes		
			Modifications of purine metabolism, with downregulated ATP and up-regulated xanthosine and hypoxanthine		
			Lower IC_50_ compared to cisplatin in vitro		
			Enhanced Pt-DNA adducts formation		
			Significant upregulation of ABAT and CLDN6 genes, with inhibition of cancer cell proliferation and apoptosis		
			Increased arginine and decreased carnitine, with apoptosis		
HSA-His242-Pt-Dp44mT NPs	HSA–Pt compound complex NPs	[Pt(Dp44mT)Cl] HSA glutaraldehyde	Enhanced inhibition of tumor growth	Developing a novel generation of Pt-based drugs Enabling multimodal therapy, and further improving drug delivery Important insights into the interaction of HSA with metal drugs but also supporting the medical background of HSA vectors	[92]
			Reduced toxicity		
			Destruction of cancer cells by inducing apoptosis, autophagy, and inhibiting angiogenesis		

## Data Availability

No new data was created in this study.

## Abbreviations

The following abbreviations are used in this manuscript:

DNA	deoxyribonucleic acid
ROS	reactive oxygen species
OS	oxidative stress
Pt	platinum
NP	nanoparticle
GSH	glutathione
ATP	adenosine triphosphate
MRP2	multidrug resistance protein 2
Cu	copper
LND	lonidamine
TPP	triphenylphosphine
HA-CD	β-cyclodextrin-grafted hyaluronic acid
mtDNA	mitochondrial deoxyribonucleic acid
Bcl-2	B-cell lymphoma 2
PEG	polyethylene glycol
OXA	oxaliplatin
STING	stimulator of interferon genes
IDOi	indoleamine-(2/3)-dioxygenase inhibitor
ALN	alendronate
ICD	immunogenic cell death
NIR-II	near-infrared-II
UVA	ultraviolet A
pAkt	phosphorylated protein kinase B
siRNA	small interfering RNA
CSNK2A1	casein kinase 2 alpha 1
siXkr8	small interfering RNA targeting XK-related protein 8
Au	gold
FMN	riboflavin-5′-phosphate
Fe	iron
OH^•^	hydroxyl radical
H_2_O_2_	hydrogen peroxide
GPX4	glutathione peroxidase 4
VEGF-A	vascular endothelial growth factor-A
MRI	magnetic resonance imaging
EGFR	epidermal growth factor receptor
Cy	1-(2-hydroxyethyl)-2-((E)-2-((E)-3-((E)-2-(1-(2-hydroxyethyl)-3,3-dimethylindolin-2-ylidene)ethylidene)-2-chlorocyclohex-1-en-1-ly)vinyl)-3,3-dimethyl-3H-indol-1-ium bromide
USP1i	ubiquitin-specific protease 1 inhibitor
S	sulfur
NSCLC	nonsmall cell lung carcinoma
NO	nitric oxide
BLZ-945	Sotuletininb
